# EEG upper/low alpha frequency power ratio relates to temporo-parietal brain atrophy and memory performances in mild cognitive impairment

**DOI:** 10.3389/fnagi.2013.00063

**Published:** 2013-10-25

**Authors:** Davide V. Moretti, Donata Paternicò, Giuliano Binetti, Orazio Zanetti, Giovanni B. Frisoni

**Affiliations:** Istituto di Ricovero e Cura a Carattere Scientifico Centro San Giovanni di Dio FatebenefratelliBrescia, Italy

**Keywords:** EEG, brain rhythms, cortical atrophy, MRI, MCI-diagnosis

## Abstract

**Objective:** Temporo-parietal cortex thinning is associated to mild cognitive impairment (MCI) due to Alzheimer disease (AD). The increase of EEG upper/low alpha power ratio has been associated with AD-converter MCI subjects. We investigated the association of alpha3/alpha2 ratio with patterns of cortical thickness in MCI.

**Materials and Methods:** Seventy-four adult subjects with MCI underwent clinical and neuropsychological evaluation, electroencephalogram (EEG) recording and high resolution 3D magnetic resonance imaging. Alpha3/alpha2 power ratio as well as cortical thickness was computed for each subject. Three MCI groups were detected according to increasing tertile values of upper/low alpha power ratio. Difference of cortical thickness among the groups was estimated. Pearson’s *r* was used to assess the topography of the correlation between cortical thinning and memory impairment.

**Results:** High upper/low alpha power ratio group had total cortical gray matter volume reduction of 471 mm^2^ than low upper/low alpha power ratio group (*p* < 0.001). Upper/low alpha group showed a similar but less marked pattern (160 mm^2^) of cortical thinning when compared to middle upper/low alpha power ratio group (*p* < 0.001). Moreover, high upper/low alpha group had wider cortical thinning than other groups, mapped to the Supramarginal and Precuneus bilaterally. Finally, in high upper/low alpha group temporo-parietal cortical thickness was correlated to memory performance. No significant cortical thickness differences was found between middle and low alpha3/alpha2 power ratio groups.

**Conclusion:** High EEG upper/low alpha power ratio was associated with temporo-parietal cortical thinning and memory impairment in MCI subjects. The combination of EEG upper/low alpha ratio and cortical thickness measure could be useful for identifying individuals at risk for progression to AD dementia and may be of value in clinical context.

## INTRODUCTION

The mild cognitive impairment (MCI) commonly represent the “at-risk” state of developing dementia. There is therefore a need for developing early biomarkers which allow to identify subjects who could develop the disease, useful for early diagnosis and effective prevention therapies. The identification and validation of biomarkers for diagnosing, monitoring progression and predicting onset of Alzheimer’s disease (AD) has been a main focus of AD research in the past 10 years. In line with recently published research criteria, it is becoming clear that the integration of different biomarkers is a milestone for a correct and early diagnosis of AD (Dubois et al., 2007; Albert et al., 2011). To date, the most studied and validated biomarkers are Abeta42 and tau protein in the cerebrospinal fluid (CSF), glucose hypometabolism on fluorodeoxyglucose positron emission tomography (18F-FDG PET), atrophy of hippocampal volume (HV) on magnetic resonance (MR), and brain amyloid deposition on amyloid imaging with PET (Hampel et al., 2008; Galluzzi et al., 2013). Anyway, some controversies remane to debate. The latter biomarkers have a good sensibility in identifying subjects with a neurodegenerative disorders at high risk to convert in dementia, but they lack a reliable specificity that allow a clear-cut diagnosis of the different subtypes of dementias. Of note, in neurodegenerative disorders, like AD or other dementias, the brain networks modifies many years before clinical manifestations. Recent MRI studies have demonstrated that a large neural network is altered in subjects with prodromal AD (Frisoni et al., 2006, 2007, 2008, 2009; van Strien et al., 2009; Frisoni, 2012). In particular, subjects with cognitive decline have shown early atrophy and loss of gray matter in cortical specific brain areas (Frisoni et al., 2007, 2009), including including precuneus, hippocampal, medial temporal, and parietal lobes. In the conceptual frame of the integration of biomarkers for an early and highly predictive diagnosis, the EEG could be a reliable tool (Missonnier et al., 2010). Indeed, it is widely accepted that the cerebral EEG rhythms reflect the underlying brain network activity (Steriade, 2006). As a consequence, modifications in EEG rhythms could be an early sign of AD. In particular, the study of alpha rhythm seems to be a very suitable tool to detect relationship between structural and functional brain networks (Lopes da Silva et al., 1980; Nunez, 1989; Stam et al., 2005; Moretti et al., 2009a,b, 2011a; Ingber and Nunez, 2011). Previous studies has convincingly demonstrated that there are thalamo-cortical and cortico-cortical components which interact in the generation of cortical alpha rhythms. Recently, it has been demonstrated that the increase of high alpha relative to low alpha power is a reliable EEG marker of hippocampal atrophy (Moretti et al., 2011b) and amigdalo-hippocampal complex atrophy (Moretti et al., 2012b). Furthermore, the increase in alpha3/alpha2 power ratio has been demonstrated predictive of conversion of patients with MCI in AD, but not in non-AD dementia (Moretti et al., 2012a). The same increase of alpha3/alpha2 power ratio was found to be correlated with hippocampal atrophy in subjects with AD (Bakkour et al., 2009). Finally, a recent study have shown that MCI subjects with highest alpha3/alpha2 power ratio present a peculiar pattern of basal ganglia and thalamic atrophy, detected with voxel-based-morphometry (VBM) technique, as compared to MCI groups with middle and low alpha3/alpha2 power ratio (Klimesch et al., 2006, 2007). The present explorative study shares the same theoretical background. The aim of the study is to extend the relationship of the alpha3/alpha2 EEG power ratio to the study of cerebral cortex atrophy in subjects with MCI. Two principal reasons supports this aim: (1) the hippocampal complex is strongly connected with temporo-parietal cortex; (2) in AD due to MCI there is the presence of both hippocampal and brain cortical atrophy. Moreover, increasing thinning in specific cortical areas is peculiar of AD and it could predicts conversion from MCI state to AD dementia (Shannon and Weaver, 1949; Folstein et al., 1975; Rosen et al., 1980; Hughes et al., 1982; Klimesch, 2011). To the best of our knowledge, an approach considering the regional pattern of cortical atrophy in combination with the EEG markers was never be investigated. This is the first study investigating the pattern of cortical thickness in a population of MCI subjects with increasing levels of alpha3/alpha2 ratios. Results show that subjects with higher a3/a2 when compared to subjects with lower and middle a3/a2 power ratio showed significant and wide thinning both of global cortical volume and specific brain areas, like the Supramarginal gyrus and Precuneus bilaterally. Other smaller regions of cortical thinning were localized on the right hemisphere in the insula, parietal, and temporal cortex.

## MATERIALS AND METHODS

### SUBJECTS

For the present study, 74 subjects with MCI was recruited from the memory Clinic of the Scientific Institute for Research and Care (IRCCS) of Alzheimer’s and psychiatric diseases “Fatebenefratelli” in Brescia, Italy. All experimental protocols had been approved by the local ethics committee. Informed consent was obtained from all participants or their caregivers, according to the Code of Ethics of the World Medical Association (Declaration of Helsinki).

### DIAGNOSTIC CRITERIA

Patients were selected from a prospective study on the natural history of cognitive impairment (the translational outpatient memory clinic – TOMC study) carried out in the outpatient facility of the National Institute for the Research and Care of Alzheimer’s Disease (IRCCS, Istituto Centro San Giovanni di Dio Fatebenefratelli, Brescia, Italy). Patients were rated with a series of standardized diagnostic and severity instruments, including the Mini-Mental State Examination (MMSE; Lawton and Brodie, 1969), the Clinical Dementia Rating Scale (CDRS; Petersen et al., 2001), the Hachinski Ischemic Scale (HIS; Portet et al., 2006) the instrumental and basic activities of daily living (IADL, BADL; Radloff, 1977; Moretti et al., 2003; Lezak et al., 2004) and a complete neuropsyshological assessment (Klimesch, 1997, 1999). All the neuropsychological tests were standardized on Italian population, thus scores were compared to normative values with age, education and gender corrections in an Italian population. In addition, patients underwent diagnostic neuroimaging procedures (magnetic resonance imaging, MRI), and laboratory testing to rule out other causes of cognitive impairment. Inclusion criteria of the study were all of the following: (i) complaint by the patient, or report by a relative or the general practitioner, of memory or other cognitive disturbances; (ii) MMSE score of 24–27/30, or MMSE of 28 and higher plus low performance (score of 2–6 or higher) on the clock drawing test (Klimesch, 1997); (iii) sparing of IADL, BADL or functional impairment steadily due to causes other than cognitive impairment, such as physical impairments, sensory loss, gait or balance disturbances, etc. Exclusion criteria were anyone of the following: (i) patients aged 90 years and older (no minimum age to participate in the study); (ii) history of depression (from mild to moderate or major depression) or juvenile-onset psychosis; (iii) history or neurological signs of major stroke; (iv) other psychiatric diseases, overt dementia, epilepsy, drug addiction, alcohol dependence; (v) use of psychoactive drugs, including acetylcholinesterase inhibitors or other drugs enhancing brain cognitive functions or biasing EEG activity; and (vi) current or previous uncontrolled or complicated systemic diseases (including diabetes mellitus), or traumatic brain injuries. All subjects were right-handed. These inclusion and exclusion criteria for MCI were based on previous seminal studies (Radloff, 1977; Moretti et al., 2003; Dubois et al., 2007). As the aim of our study was to evaluate the relationship between GM loss and alpha2/alpha3 ratios in MCI subjects, we did not consider the clinical subtype of MCI, i.e., amnesic, or non-amnesic, single or multiple domains. Demographic and cognitive features of the subjects in study are summarized in **Table 1**. There were no statistical difference in age, gender and education among the groups in study

**Table 1:** Demographic and cognitive characteristics in the whole sample, disaggregated for increased levels of alpha3/alpha2 Numbers denote mean ± standard deviation, number and [range].

	Alpha3/alpha2			
	**High**	**Middle**	**Low **	***p***
**Demographic and clinical futures**				
Number of subjects	18	38	18	–
Age, years	70.4 ± 6.7 [60–85]	68.4 ± 8.2 [52–83]	70.4 ± 7.4 [57–80]	.55
Sex, female	13 (%)	24 (%)	14 (%)	.51
Education, years	6.6 ± 3.6 [4–18]	7.6 ± 3.7 [3–17]	8.3 ± 4.7 [3–18]	.42
Mini Mental State Exam	27 ± 1.7 [23–29]	27.4 ± 1.3 [24–30]	26.9 ± 1.2 [23–30]	.46
WMHs (mm^3^)	2.78 ± 2.58	5.59 ± 6.60	2.57 ± 2.76	.09
Alpha3/alpha2	1.29 ± 0.1 [1.17–1.52]	1.08 ± 0.0 [1–1.16]	0.9 ± 0.1 [0.77–0.98]	**.000****
**Memory**				
Babcock	9.6 ± 3.5	9.8 ± 4.1	9.9 ± 3.8	.99
AVLT. immediate recall	42.7 ± 7.6	38.8 ± 11.8	40.5 ± 12.8	.53
AVLT. delayed recall	8.6 ± 3.2	8.2 ± 4.1	9.1 ± 4.4	.80
Rey’s figure. recall	15 ± 6.9	12.6 ± 6.1	15 ± 7	.35******
**Language**				
Fluency phonetic	31.9 ± 7.4	33.1 ± 9.8	34.1 ± 9.5	.80
Fluency semantic	36.1 ± 6.4	33.7 ± 8.3	34.1 ± 9.8	.62
Token test	31.1 ± 2.4	32.2 ± 2.4	33.1 ± 2	.06******
**Constructional and visuo-spatial abilities**				
Rey’s figure copy	31.7 ± 5.3	31.4 ± 7.8	29.1 ± 9.9	.58******
**Attention and executive functions**				
Trail Making Test A	39.4 ± 21.5	37.1 ± 23.6	65 ± 51	.20
Trail Making Test B	117.1 ± 77.5	93 ± 77.1	120.7 ± 99.4	.48
**Non verbal reasoning**				
Raven test	24.8 ± 3.8	26.3 ± 5.8	26.9 ± 4.5	.44

*p denotes significance on ANOVA.*

### EEG RECORDINGS

The EEG activity was recorded, continuously from 19 sites by using electrodes set in an elastic cap (Electro-Cap International, Inc.) and positioned according to the 10–20 international systems (Fp1, Fp2, F7, F3, Fz, F4, F8, T3, C3, Cz, C4, T4, T5, P3, Pz, P4, T6, O1, and O2). The patients were instructed to stay sit with closed eyes and relaxed. In order to keep constant the level of vigilance, an operator controlled on-line the subject and the EEG traces, alerting the subject any time there were signs of behavioral and/or EEG drowsiness. The ground electrode was placed in front of Fz. The left and right mastoids served as reference for all electrodes. The recordings were used off-line to re-reference the scalp recordings to the common average. Re-referencing was done prior to the EEG artifact detection and analysis. Data were recorded with a band-pass filter of 0.3–70 Hz, and digitized at a sampling rate of 250 Hz (BrainAmp, BrainProducts, Germany). Electrodes-skin impedance was set below 5 khz. Horizontal and vertical eye movements were detected by recording the electrooculogram (EOG). The recording lasted 5 min, with subjects with closed eyes. Longer recordings would have reduced the variability of the data, but they would also have increased the possibility of slowing of EEG oscillations due to reduced vigilance and arousal. EEG data were then analyzed and fragmented off-line in consecutive epochs of 2 s, with a frequency resolution of 0.5 Hz. We are confident about the stationarity of EEG signal in our traces. Our recordings were performed at rest state, without any external stimulation that could bias the signal, maintaining the stochastic nature of spontaneous ongoing EEG. Moreover, it is widely accepted that the duration of a so-called quasi-stationary interval of continuous EEG recordings is expected not to exceed 2–4 s (Kaplan, 1999), but some authors found much longer fragments of even 12 s (Cohen and Sances, 1977), 25 s (Kawabata, 1976) or 40–60 s (McEwen and Anderson, 1975) to be approximately stationary. Of note, our spectral analysis has been evaluated on two-seconds epoch in each subject. Finally, the spectral power was averaged across all electrodes to a obtain a sort of global field power, which would have reduced the channel to channel variability, with the advantage to extract a high stationary measure and to obtain a smoother, clearer and homogeneous individual alpha peak. Recently, it has been demonstrated that the analysis of the EEG recording in frequency domain (i.e., power spectra) results in high stationary signal (Kipiñski et al., 2011). Anyway as a control analysis, the stability of the EEG signal was tested as follows: the power spectra of ten epochs lasting 2 s each were averaged both at the beginning at the end of the free-artifact EEG trace of any subjects, since testing for stationarity of the variability of trial-to-trial power spectra required equal time intervals between consecutive observations (Kipiñski et al., 2011). ANOVA analysis were performed showing no statistical difference (*p* = 0.2) between the beginning and the ending epochs in each subjects and among all subjects. The average number of epochs analyzed was 140, ranging from 130 to 150. The epochs with ocular, muscular and other types of artifacts were discarded by two skilled electroencephalographists (Moretti et al., 2004). The spectral power we obtained is an estimation of a spectrum collapsed all over the scalp electrodes. In this way, the eventual contribution of the muscular artifact is strongly reduced. It should be possible to compute a more focused field, chosing a subset of electrodes next to the brain region of interest. Anyway, this procedure has two main disadvantages: (1) it needs a larger array of electrodes, given the volume conduction phenomenon. Of note, a larger electrode array should require the application of some further computation like, for example, Laplacia filter or spline interpolation; this aspect is of particular importance because it is more time-consuming and not applicable in a clinical context; (2) the power computation on a smaller number of electrodes could give raise to artifactual detection of the individual alpha frequency (IAF) peak as the presence of double peak or the absence of a clear peak. This computation errors are overlooked by the power spectra computation collapsed on the whole array of electrodes. Moreover, two skilled electroencephalographists checked separately the data and after they have a common revision. No automatic methods were used. We are confident that this control procedure could prevent that artifactual EEG segments could be inserted in the analysis.

### ANALYSIS OF INDIVIDUAL FREQUENCY BANDS

All recordings were obtained in the morning with subjects resting comfortably. Vigilance was continuously monitored in order to avoid drowsiness. A digital FFT-based power spectrum analysis (Welch technique, Hanning windowing function, no phase shift) computed – ranging from 2 to 45 Hz – the power density of EEG rhythms with a 0.5 Hz frequency resolution. Two anchor frequencies were selected according to the literature guidelines (Moretti et al., 2007, 2008), that is, the theta/alpha transition frequency (TF) and the IAF peak. IAF and TF were computed for each subject in the study. These anchor frequencies were computed on the power spectra averaged across all recording electrodes. This “collapsed spectrum method” allows to identify a robust and reliable IAF, being a normalized scalp spectrum. Finally, given that we were interested in the resting state spectral content, the alpha band and IAF were determined on the eyes closed period. There is a large body of literature showing that in older healthy people as well as in patients affected by brain degenerative disorders the standard range frequency found in young and healthy people is not applicable due to the effect of both age and disease (Cabeza, 2002; Watson et al., 2012; Balsters et al., 2013). As a consequence, the computation of an IAF peak is mandatory in our study. Recent studies have convincingly shown that the IAF is very reliable in rest condition EEG recording (Grandy et al., 2013a,b; Tenke et al., 2013). Anyway, more caution has been suggested when the subjects in study have to perform some specific tasks (Bekhtereva et al., 2013). The TF marks the TF between the theta and alpha bands, and represents an estimate of the frequency at which the theta and alpha spectra intersect. TF was computed as the minimum power in the alpha frequency range, since our EEG recordings were performed at rest. The IAF represents the frequency with the maximum power peak within the extended alpha range (5–14 Hz). Based on TF and IAF, we estimated the frequency band range for each subject, as follows: delta from TF-4 to TF-2, theta from TF-2 to TF, low alpha band (alpha1 and alpha2) from TF to IAF, and high alpha band (or alpha3) from IAF to IAF + 2. The alpha1 and alpha2 bands were computed for each subject as follows: alpha1 from TF to the middle point of the TF-IAF range, and alpha2 from such middle point to the IAF peak. Moreover, individual beta and gamma frequencies were computed. Three frequency peaks were detected in the frequency range from the individual alpha3 frequency band and 45 Hz. These peaks were named beta1 peak (IBF 1), beta2 peak (IBF 2) and gamma peak (IGF). Based on peaks, the frequency ranges were determined. Beta1 ranges from alpha3 to the lower spectral power value between beta1 and beta2 peak; beta2 frequency ranges from beta1 to the lower spectral power value between beta2 and gamma peak; gamma frequency ranges from beta2 to 45 Hz, which is the end of the range considered. Moreover, within theta frequency the frequency peak (individual theta frequency, ITF) was also individuated. The mean frequency range computed in MCI subjects considered as a whole are: delta 2.9–4.9 Hz; theta 4.9–6.9 Hz; alpha1 6.9–8.9 Hz; alpha2 8.9–10.9 Hz; alpha3 10.9–12.9 Hz; beta1 12.9–19.2 Hz; beta2 19.2–32.4; gamma 32.4–45. Finally, in the frequency bands determined on an individual basis, we computed the relative power spectra for each subject. The relative power density for each frequency band was computed as the ratio between the absolute power and the mean power spectra from 2 to 45 Hz. The relative band power at each band was defined as the mean of the relative band power for each frequency bin within that band. The alpha3/alpha2 was computed in all subjects and three groups were obtained according to increasing tertiles values of alpha3/alpha2: low (a3/a2 < 1); middle (1 < a3/a2 < 1.16) and high (a3/a2 > 1.17). The tertile division allows a balanced distribution of the study samples with the advantage to avoid the extreme value in the statistical analysis. The three groups of MCI has been demonstrated in previous studies to be different in nature. In particular, the high alpha3/alpha 2 EEG power ratio MCI group is at major risk to convert to AD (Moretti et al., 2012a), as well as to have different pattern of hippocampal atrophy (Klimesch et al., 2006) and basal ganglia and thalamus gray matter lesions (Klimesch et al., 2007) as compared to the other alpha3/alpha2 power ratio MCI groups. Moreover, this group subdivision has been chosen for reason of homogeneity and comparability with the previous studies.

### MRI SCANS

For each subject, a high-resolution sagittal T1 weighted volumetric MR scan was acquired at the Neuroradiology Unit of the “Citta` di Brescia” Hospital, Brescia, by using a 1.0 T Philips Gyroscan scanner, with a gradient echo 3D technique: TR = 20 ms, TE = 5 ms, flip angle = 30, field of view = 220 mm, acquisition matrix 256 · 256, slice thickness 1.3 mm.

### CORTICAL THICKNESS ESTIMATION STEPS

Cortical thickness measurements for 74 MCI patients were made using a fully automated MRI-based analysis technique: FreeSurfer v5.1.0, a set of software tools for the study of cortical and subcortical anatomy. Briefly, in the cortical surface stream, the models of the boundary between white matter and cortical gray matter as well as the pial surface were constructed. Once these surfaces are known, an array of anatomical measures becomes possible, including: cortical thickness, surface area, curvature, and surface normal at each point on the cortex. In addition, a cortical surface-based atlas has been defined based on average folding patterns mapped to a sphere and surfaces from individuals can be aligned with this atlas with a high-dimensional nonlinear registration algorithm. The surface-based pipeline consists of several stages previous described in detail (Ségonne et al., 2004; Bekhtereva et al., 2013).

#### Single subject analysis

For each subjects the T1-weighted, anatomical 3-D MRI dataset were converted from Dicom format into .mgz format, then intensity variations are corrected and a normalized intensity image is created. The volume is registered with the Talairach atlas through an affine registration. Next, the skull is stripped using a deformable template model (Fischl and Dale, 2000) and extracerebral voxels are removed. The intensity normalized, skull-stripped image is then operated on by a segmentation procedure based on the geometric structure of the gray–white interface. Voxels are classified as white or gray matter, cutting planes are chosen to separate the hemispheres from each other. A white matter surface is then generated for each hemisphere by tiling the outside of the white matter mass for that hemisphere. This initial surface is then refined to follow the intensity gradients between the white and gray matter. The white surface is then nudged to follow the intensity gradients between the gray matter and CSF, obtaining the pial surface. Cortical thickness measurements were obtained by calculating the distance between those surfaces (white and pial surface) at each of approximately 160,000 points per hemisphere across the cortical mantle (Han et al., 2006).

#### Group analysis

In order to relate and compare anatomical features across subjects, it is necessary to establish a mapping that specifies a unique correspondence between each location in one brain and the corresponding location in another. Thus, the pial surface of an individual subject is inflated to determine the large-scale folding patterns of the cortex and subsequently transformed into a sphere to minimize metric distortion. The folding patterns of the individual are then aligned with an average folding pattern using a high-resolution surface-based averaging. Thickness measures were mapped to the inflated surface of each participant’s brain reconstruction allowing visualization of data across the entire cortical surface. Finally, cortical thickness was smoothed with a 20-mm full width at half height Gaussian kernel to reduce local variations in the measurements for further analysis.

### TEST-RETEST REPRODUCIBILITY OF CORTICAL THICKNESS ANALYSIS

Previous studies have been investigated the reliability of the cortical thickness measurements: some of these addressed the effect of scanner-specific parameters, including field strength, pulse sequence, scanner upgrade, and vendor. The use of a different pulse sequence had a larger impact, as did different parameters employed in data processing. The within-scanner variability of global cortical thickness measurements reported in previous studies was 0.03–0.07 in average (DeCarli et al., 2005; Pennanen et al., 2005; Gronenschild et al., 2012). Scanner upgrade did not increase variability nor introduce bias while measurements across field strength were slightly biased (thicker at 3 T). In the study by Han et al. (2006) and Gronenschild et al. (2012) the variability was 0.15 and 0.17 mm in average, respectively, for cross-scanner (Siemens/GE) and cross-field strength (1.5 T/3 T) comparisons. The recent study by Gronenschild et al. (2012) also investigated the effects of data processing conditions such as FreeSurfer version, workstation, and Macintosh operating system version. The authors reported significant differences between FreeSurfer version (average 2.8–3%) and a smaller differences between workstation and operating system version. On the whole, the results suggest that MRI-derived cortical thickness measures are highly reliable, however, it is important to keep consistent the MRI parameters and data processing factors within any given structural neuroimaging study.

### WHITE MATTER HYPERINTENSITIES COMPUTATION

White matter hyperintensities (WMHs) segmentation was performed on the FLAIR sequences using previously described algorithms [57. Briefly, the procedure includes (i) filtering of FLAIR images to exclude radiofrequency inhomogeneities; (ii) segmentation of brain tissue from CSF; (iii) modeling of brain intensity histogram as a Gaussian distribution; and (iv) classification of the voxels whose intensities were 3.5 standard deviations (SDs) above the mean as WMHs. In order to place each subject’s WMHs map onto a common template, a set of linear and nonlinear transformations were applied with the protocol described below. FLAIR images were coregistered to the high resolution T1 image using a 9-parameters affine transformation (i.e., including rotation, translation and scaling) and the same alignment parameters were applied to the WMHs mask image created at the previous step. T1 images were intensity corrected in order to reduce adverse impact of the WMH voxel values on the accuracy of the nonlinear warping algorithm (McKhann et al., 2011). This step involved estimating the normal white matter mean intensities surrounding the voxels classified as WMHs and replacing voxels in the T1 image corresponding to WMHs by the estimated values. T1 intensity-corrected images were normalized onto a standard template by mean of high-dimensional cubic B-spline warp (Sperling et al., 2011). The template image used in this study was created from the MRI scans of a population of elderly subjects with a mix of diagnosis (AD, MCI, and normal) and was defined as the one that minimize the amount of distortion necessary to non-linearly align each subject MRI of the population. The parameters computed from nonlinear warping were then used to warp each subject’s FLAIR image and WMHs mask onto the template (Markesbery et al., 2006). At the end of the process, anatomical regions are accurately matched between subjects, and WMHs voxels are in locations analogs to their original locations in the subjects’ image. Total vascular load was then computed for each subject by counting the number of voxels segmented as WMHs and multiplying by voxel size (1.44 mm^3^). The WMHs were estimated to exclude significant differences in the cerebrovascular load in MCI groups and to avoid that EEG results was due to cerebrovascular pathology and not to degenerative background. Moreover, in order to avoid confounding variables the WMHs were computed as covariates in statistical analysis. Of note, it has been demostrated that the vascular load could bias the brain rhythms computation (Cabeza, 2002; Moretti et al., 2008, 2011b).

### STATISTICAL ANALYSIS

Differences between groups in sociodemographic and neuropsychological features were analyzed using SPSS version 13.0 (SPSS, Chicago, IL, USA) performing an analysis of variance (ANOVA) for continuous variables and paired χ^2^ test for dichotomous variables. For continuous variables, post-hoc pairwise comparisons among groups were performed with the Games-Howell or Bonferroni tests depending on homogeneity of variance tested with Levene’s test.

Concerning the neuroimaging analysis, the Qdec interface in Freesurfer software was used: a vertex-by-vertex analysis was carried out performing a general linear model to analyze whether any difference in mean cortical thickness existed between groups (low: a3/a2 < 1 μV^2^; middle: 1 < a3/a2 < 1.16 μV^2^; high: a3/a2 > 1.17 μV^2^). The following comparisons were carried out: high vs. low, high vs. middle and middle vs. low. Age, sex, education, global cognitive level (MMSE score) and WMHs were introduced as covariates in the analysis to avoid confounding factors. We first tried to apply an appropriate Bonferroni multiple-comparison correction in our analysis (at *p* < 0.05 corrected). Unfortunately no *p*-value survived after this correction. Thus we choose to set a more restrictive significance threshold (than *p* < 0.05 corrected) at *p* < 0.001 uncorrected for multiple comparison. Moreover, we considered as significant only the clusters which also were wide equal or major to 30 mm^2^. Finally a surface map was generated to display the results on an average brain. For illustrative purpose significance was set to a *p*-value of í0.01 uncorrected for multiple comparisons.

As a control analysis, in order to exclude casual relationships between EEG markers and cortical volumes, a correlation between brain areas and memory performance has been studied. The correlation analysis was performed on the three samples separately (high a3/a2, low a3/a2, middle a3/a2) and on the entire sample (high and low and middle grouped together). An exploratory analysis of non-linear correlation does not fit into purpose of testing our a priori hypothesis. Indeed we choose to apply a measure of linear dependence led by our a priori hypotheses for which the MCI group with the greater cortical thinning, and higher a3/a2 EEG level (indicating an incipient AD) should show a clear correlation with the memory tests performance, in the sense that an increase in cortical thinning corresponds to a decrease in memory performance, and vice versa. Indeed, even if in the cognitive tests scores there are no significant differences, we hypothesized that the MCI group with the greater cortical thinning, and higher a3/a2 EEG level, indicating an incipient AD, should show a clear correlation with the memory tests performance. The correlation analysis on a vertex-by-vertex basis was performed specifically for the following neuropsychological memory test results: Babcok test, Rey auditory verbal learning test (AVLT) immediate recall, and Rey AVLT delayed recall. The analysis was thresholded at *p* < 0.001 uncorrected for multiple comparisons while results were mapped at *p*-value of <0.005 uncorrected for illustrative purpose. Only the clusters which survived at the statistical threshold and wide equal or major to 15 mm^2^ were considered as significant.

## RESULTS

**Table 1** shows the sociodemographic and neuropsychological characteristics of MCI subgroups defined by the tertile values of alpha3/alpha2. The ANOVA analysis showed that there was not statistically significant differences between groups which resulted well paired for age, sex, WMHs burden, education and global cognitive level. Anyway, age, sex, education, global cognitive level (MMSE score) and WMHs were introduced as covariates in the subsequent analysis to avoid confounding factors. Alpha3/alpha2 ratio levels were significant at Games-Howell post hoc comparisons (*p* = 0.000).

### PATTERN OF CORTICAL THICKNESS BETWEEN GROUPS

**FIGURE 1:**
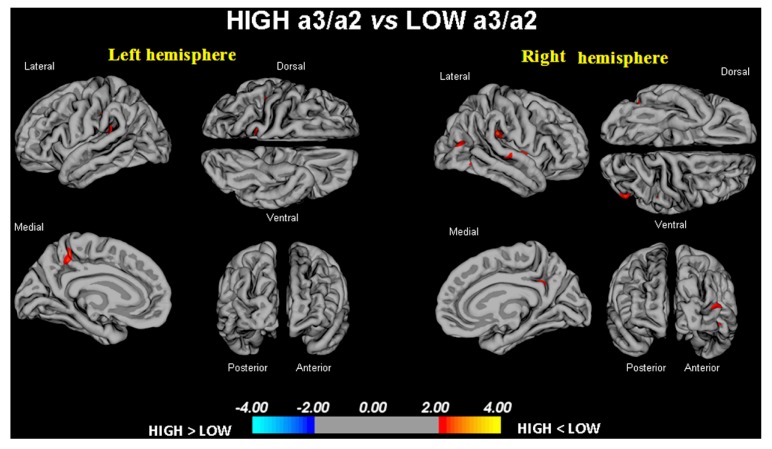
**In red are represented the brain regions with higher regional cortical thickness in MCI with high a3/a2 ratio as compared to MCI with low a3/a2 ratio (*p* < 0.01 uncorrected).** The color-coding for *p* values is on a logarithmic scale. Results are presented on the pial cortical surface of brain: dark gray regions represent sulci and light gray regions represent gyri.

High vs. low: when compared to subjects with low a3/a2 ratios, patients with high a3/a2 ratio show thinning in the bilateral SuperioTemporal, Supramarginal and Precuneus cortex, in the right Inferior Parietal and Insula. The total CGM reduction in *high* a3/2 group than *low* a3/a2 group was 471 mm^2^ (**Figure 1**; **Table 2**).

**Table 2:** Brain regions with significant regional cortical thickness differences in MCI with high a3/a2 ratio compared to MCI with low a3/a2 ratio (high a3/a2 < low a3/a2) and MCI with middle a3/a2 ratio (high a3/a2 < middle a3/a2).

Cluster size (mm^2^)	Region	Side	Stereotaxic coordinate			*P*	Thickness (mm^2^)	
			*x*	*y*	*z*		High	Low
**High a3/a2 < low a3/a2**								
61	Superior temporal	L	-47	-36	9	<0.0001	2.25 ± 0.15	2.00 ± 0.18
60	Supramarginal	L	-40	-36	18	0.0008	1.95 ± 0.18	2.08 ± 0.19
35	Precuneus	L	-14	-48	58	0.0002	1.87 ± 0.14	1.98 ± 0.19
58	Superior temporal	R	61	-22	-1	<0.0001	2.18 ± 0.17	2.24 ± 0.24
59	Supramarginal	R	49	-29	27	<0.0001	1.94 ± 0.18	2.08 ± 0.20
52	Precuneus	R	11	-49	30	0.0001	1.81 ± 0.17	1.93 ± 0.15
85	Inferior parietal	R	46	-75	10	0.0001	1.94 ± 0.22	2.07 ± 0.23
61	Insula	R	38	-2	3	0.0002	2.37 ± 0.28	2.57 ± 0.36
**High a3/a2 < middle a3/a2**								
59	Postcentral	L	-57	-18	18	0.0002	1.51 ± 0.15	1.62 ± 0.17
71	Supramarginal	L	-53	-42	46	0.001	1.95 ± 0.18	2.11 ± 023
**33**	Precuneus	L	-16	-43	60	0.001	1.87 ± 0.14	1.94 ± 0.17

*Cluster size represents the extension of contiguous significant voxels in the cluster obtained at *p* < 0.01 uncorrected (cluster size > 30 mm^2^). Stereotaxic coordinates reveal the position of the most significant voxel of the cluster, and side denotes its localization on the left (L) or right (R) brain hemisphere. Thickness denotes the average cortical thickness and standard deviation values within the cluster in high and middle a3/a2 groups. *P* denotes the significance level of the differences in thickness between groups.*

**FIGURE 2:**
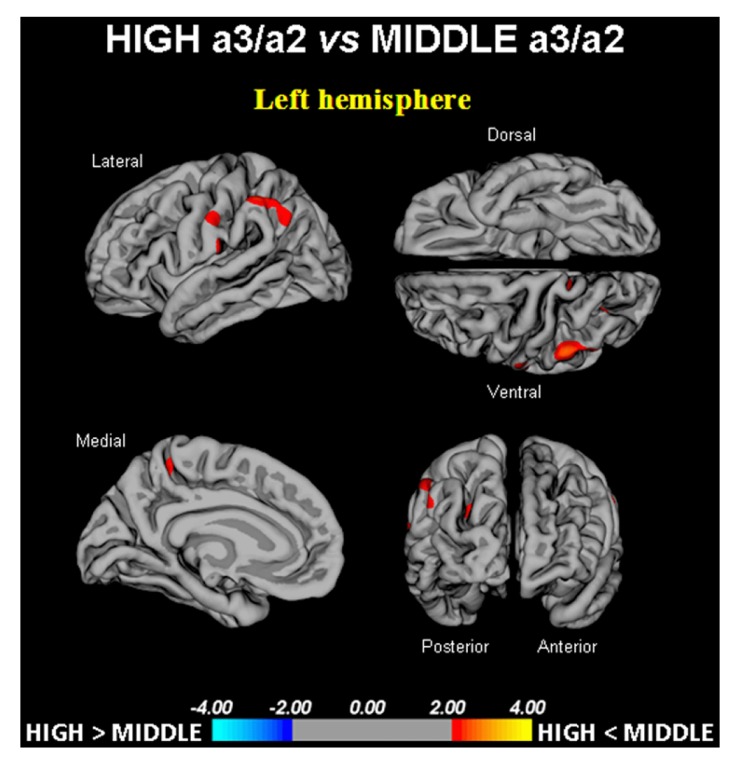
**In red are represented the brain regions with higher regional cortical thickness in MCI with high a3/a2 ratio as compared to MCI with middle a3/a2 ratio (*p* < 0.01 uncorrected).** The color-coding for *p* values is on a logarithmic scale. Results are presented on the pial cortical surface of brain: dark gray regions represent sulci and light gray regions represent gyri.

High vs. middle: the same group showed a similar but less wide pattern of cortical thinning when compared to middle a3/a2 group: the regions of atrophy were located in the left Supramarginal gyrus, left Precuneus and Postcentral cortex. The total CGM reduction in High a3/2 group than middle a3/a2 group was 160 mm^2^ (**Figure 2**; **Table 2**). When High group was compared to low group the total extension of cortical thinning (471 mm^2^) was 34% wider than the other comparison in which High group was compared to middle group (160 mm^2^). No regions of major cortical atrophy was found in groups with middle or low a3/a2 power ratio when compared to High a3/a2 group. No significant cortical thickness differences was found between middle and low a3/a2 groups.

### CORRELATIONS BETWEEN NEUROPSYCHOLOGICAL MEMORY TESTS AND CORTICAL THICKNESS IN HIGH A3/A2 GROUP AND OTHER GROUPS

**FIGURE 3:**
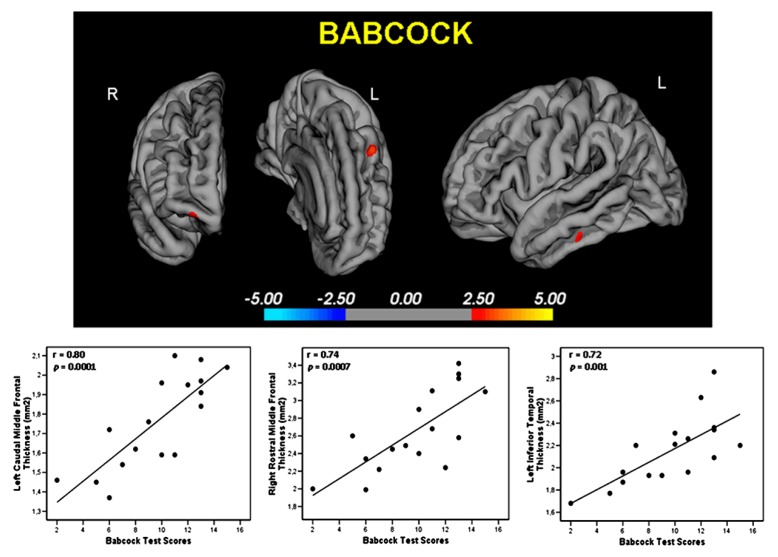
**Regions with significant correlation between memory performance evaluated with the Babcock Test and cortical thickness measure in the High a3/a2 group.** *r* and *p* denote the correlation coefficient and significance level respectively.

Babcock test: a significant positive correlation was found in High alpha3/alpha2 group between logical memory performance at Babcock test and thickness values in the left caudal middle frontal (cluster size = 36 mm^2^; stereotaxic coordinate *x, y, z* = -34, 22, 47; *r* = 0.80; *p* = 0.0001) and left Inferior Temporal (15 mm^2^; -54, -28, -26; *r* = 0.72; *p* = 0.001), right rostral middle frontal (28 mm^2^; 2^3^, 56, -13; *r* = 0.74; *p* = 0.0007; **Figure 3**). No significant correlation was found with the same regions nor in the other groups nor in the whole sample (see also Table in Supplementary materials).

**FIGURE 4:**
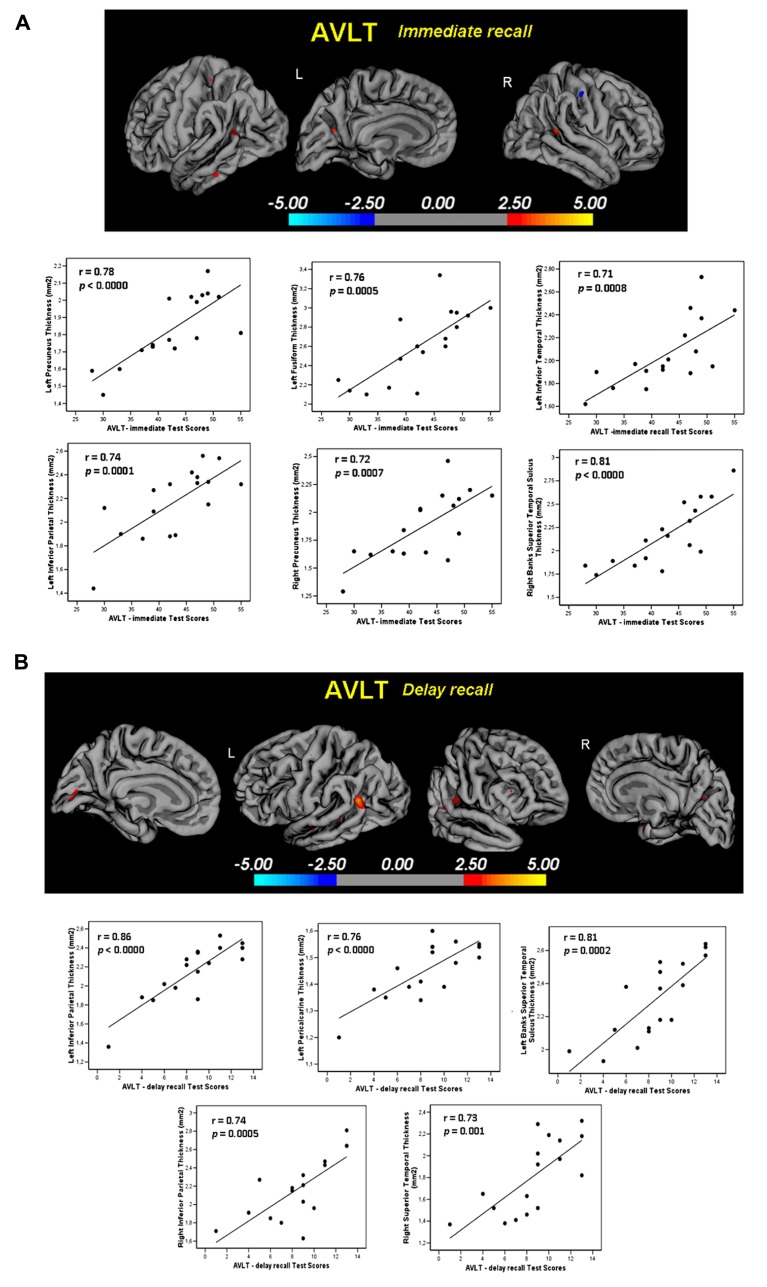
**(A)** Regions with significant correlation between memory performance evaluated with the AVLT Test- immediate recall- and cortical thickness measure in the High a3/a2 group. *r* and *p* denote the correlation coefficient and significance level respectively. **(B)** Regions with significant correlation between memory performance evaluated with the AVLT Test -delay recall- and cortical thickness measure in the High a3/a2 group. *r* and *p* denote the correlation coefficient and significance level respectively.

AVLT immediate recall: in High alpha3/alpha2 group memory performance were significant related with the cortical thickness values in the bilateral precuneus (left: 47 mm^2^; -21, -61, 20; *r* = 0.78; *p* < 0.0000; right: 58 mm^2^; 20, -60, 25; *r* = 0.72; *p* = 0.0007), left fusiform (40 mm^2^; -41, -25, -21; *r* = 0.76; *p* = 0.0005), inferior parietal (43 mm^2^; -46, -60, 11; *r* = 0.74; *p* = 0.0001), inferior temporal (35 mm^2^; -53, -34, -21; *r* = 0.71; *p* = 0.0008), and right Banks of the superior temporal sulcus (44 mm^2^; 48, -48, 9; *r* = 0.81; *p* < 0.000; **Figure 4A**). Memory performance was correlated in the middle group with both the right precuneus also (*r* = 0.19 and *p* = 0.03), and the right Banks of the superior temporal sulcus (*r* = 0.44, *p* = 0.02). No significant associations was find either in the low group nor in the entire sample (**Table 3**).

**Table 3:** Correlations between neuropsychological memory tests and cortical thickness in High a3/a2 group and other groups, including whole sample group.

	Regions	High (*n* = 18)	Middle (*n* = 38)	Low (*n* = 18)	ALL (*n* = 74)
Babcock	Left caudal middle frontal	*r* = 0.80	*r* = 0.10	*r* = 0.24	*r* = 0.18
		*p* = 0.0001*	*p* = 0.58	*p* = 0.37	*p* = 0.18
	Right rostral middle frontal	*r* = 74	*r* = 0.08	*r* = 0.37	*r* = 0.16
		*p* = 0.0007*	*p* = 0.69	*p* = 0.16	*p* = 0.21
	Left inferior temporal	*r* = 0.72	*r* = 0.08	*r* = 0.07	*r* = 0.13
		*p* = 0.001*	*p* = 0.69	*p* = 0.80	*p* = 0.31
	Left precuneus	*r* = 0.78	*r* = 0.25	*r* = ?0.73	*r* = 0.18
		*p* < 0.0000*	*p* = 0.18	*p* = 0.79	*p* = 0.18
	Left fusiform	*r* = 0.76	*r* = 0.21	*r* = ?0.08	*r* = 0.16
		*p* = 0.0005*	*p* = 0.26	*p* = 0.76	*p* = 0.22
AVLT immediate	Left inferior temporal	*r* = 0.71	*r* = 0.29	*r* = ?0.10	*r* = 0.18
		*p* = 0.0008*	*p* = 0.12	*p* = 0.70	*p* = 0.17
	Left inferior parietal	*r* = 0.74	*r* = 0.28	*r* = 0.12	*r* = 0.21
		*p* = 0.001*	*p* = 0.14	*p* = 0.67	*p* = 0.09
	Right precuneus	*r* = 0.72	*r* = 0.19	*r* = 0.02	*r* = 0.21
		*p* = 0.0007*	*p* = 0.03*	*p* = 0.38	*p* = 0.09
	Righ banks of the superior temporal sulcus	*r* = 0.81	*r* = 0.44	*r* = 0.04	*r* = 0.31
		*p* < 0.0000*	*p* = 0.02*	*p* = 0.38	*p* = 0.01*
	Left inferior parietal	*r* = 0.86	*r* = 0.25	*r* = ?0.05	*r* = 0.18
		*p* < 0.0000*	*p* = 0.18	*p* = 0.87	*p* = 0.15
	Left pericalcarine	*r* = 0.76	*r* = 0.15	*r* = 0.69	*r* = 0.17
		*p* < 0.0000*	*p* = 0.42	*p* = 0.80	*p* = 0.17
AVLT delayed	Left banks of the superior temporal sulcus	*r* = 0.81	*r* = 0.24	*r* = ?0.15	*r* = 0.12
		*p* = 0.0002*	*p* = 0.20	*p* = 0.58	*p* = 0.34
	Right inferior parietal	*r* = 0.74	*r* = 0.19	*r* = 0.03	*r* = 0.16
		*p* = 0.0005*	*p* = 0.31	*p* = 0.92	*p* = 0.21
	Right superior temporal	*r* = 0.73	*r* = 0.20	*r* = 0.16	*r* = 0.21
		*p* = 0.001*	*p* = 0.30	*p* = 0.56	*p* = 0.09

AVLT delayed recall: in High alpha3/alpha2 group memory function correlate significantly with cortical thickness in the bilateral Inferior Parietal (left: 95; -44, -58, 12; *r* = 0.86; *p* < 0.0000; right: 49; 50, -50, 9; *r* = 0.74; *p* = 0.0005), left Pericalcarine cortex (54; -7, -8, 11; *r* = 0.76; *p* < 0.0000) and Banks of the Superior Temporal Sulcus (31; -51, -41, -5; *r* = 0.81; *p* = 0.0002); in the right Superior Temporal (22; 56, -34,-13; *r* = 0.73; *p* = 0.001; **Figure 4B**). No significant correlation was found with the same regions nor in the other groups nor in the whole sample (**Table 3**).

## DISCUSSION

### ASSOCIATION BETWEEN EEG MARKERS AND GM CHANGES

In the present study the relationship between an EEG marker (the alpha3/alpha2 power ratio) and the cortical thickness in subjects with MCI was investigated. The alpha3/alpha 2 power ratio has been chosen because in previous works it has been demonstrated that MCI subjects with higher alpha3/alpha2 ratio are at major risk to develop AD (Klimesch et al., 2006, 2007; Bakkour et al., 2009; Moretti et al., 2012a). Our results show that the MCI group with higher alpha3/alpha2 ratio has a greater global cortical atrophy than the other subgroups, thus confirming a large body of literature (Frisoni et al., 2007; Moretti et al., 2012a). Furthermore, the greater atrophy is significant in two specific brain areas: precuneus and supramarginal gyrus (a brain area belonging to the inferior parietal lobule), both in left and right hemisphere. These results was largely expected considering previous studies. Indeed, structural and functional abnormalities of the precuneus were observed in MCI (Matsuda, 2007; Petrella et al., 2007; Dai et al., 2009; Pihlajamaki et al., 2009) as well a in Azheimer’s disease (Dickerson and Sperling, 2009; Ryu et al., 2010; Sperling et al., 2010) so that the atrophy of precuneus has been considered as a pathognomonic marker of early AD. Recent studies suggest that specific regions, namely the precuneus and posterior cingulate, together with the medial temporal lobe, are selectively vulnerable to early amyloid deposition in AD pathology (Pievani et al., 2011; de Haan et al., 2012).

### NEUROPHYISIOLOGICAL AND CLINICAL IMPLICATIONS

Recent studies have demonstrated that during the successful encoding of new items there is a desynchronization in the temporo-parietal memory-related networks whereas a synchronization prevent a successful semantic encoding (Sperling et al., 2010; Chatwal and Sperling, 2012). The deleterious role of synchronization has been recently demonstrated by an interesting study facing the intriguing relationship between functional and structural degeneration in AD (de Haan et al., 2012). The authors detected some hub regions (heteromodal associative regions) selectively vulnerable in AD pathology, due to the damage of inhibitory interneurons providing a loss of inhibition at cellular level. According to the authors, the disinhibition provokes an increasing amount of neural activity at network level, giving as a final result an hypersynchronization of brain areas. Of note, this overactivity is excitotoxic and determines cellular apoptosis and brain atrophy. Also, Palop and Mucke emphasize the role of inhibitory interneuron dysfunction, leading to hypersynchronization (Stam et al., 2003; Palop and Mucke, 2010; Jones et al., 2011; Brier et al., 2012). Our results are in line with these previous influential studies. A possible integrative view of all the results could be as follows: (1) the higher neuronal activity in the hub regions starts from a disfunction of cellular inhibition; (2) the consequent disinhibition drives neural network to an oversynchronization; (3) this oversynchronization is peculiar of the hub regions with higher amyloid burden; (4) these overactivated regions are prone to degeneration and atrophy; (5) a possible neurophsyiologic sign of this oversynchronization is the increase of the alpha3/alpha 2 power ratio we have found in typical hub regions (Rossini et al., 2008; Wonderlick et al., 2009; Bhattacharya et al., 2011; Wu et al., 2011). It is of great interest that there is an overlapping between the brain regions associated with increase of EEG alpha3/alpha2 power ratio (hypersynchronization of upper alpha) in our study and the regions associated to higher amyloid burden related to memory processes (Jones et al., 2011; Brier et al., 2012). Moreover, in the present study, there is a very interesting result. The atrophy of precuneus is coupled with the atrophy in supramarginal gyrus and, at lesser extent, with inferior parietal, insula and superior temporal gyrus. This atrophy pattern is clearly expressed in the group of MCI subjects with higher alpha3/alpha2 power ratio. This finding fits well with the results of a recent study (Zhang and Li, 2012), investigating the functional connectivity of human precuneus by resting state fMRI. The authors found that there is a preferential pathway of connectivity of the dorsal precuneus with supramarginal gyrus, parietal cortex, superior temporal gyrus and insula. As a consequence, the atrophy we detected in the MCI group with higher alpha3/alpha2 ratio power could be hypothesized as the loss of GM in an entire anatomo-functional network more than atrophy of isolated brain areas. Of note, it is widely accepted that AD is the result of a cortical network impairment more than the atrophy of single cortical areas (Morbelli et al., 2012). In subjects with low or middle alpha3/alpha2 power ratio the cognitive impairment is possibly due to cerebro-vascular impairment or non-AD degenerative process. Although the rigid selection criteria adopted to include in the study patients with primary cognitive deficits, in the clinical practice is not infrequent to have MCI subjects not due to AD.

### MEMORY PERFORMANCE AND CORTICAL THINNING

In order to exclude a random relationship between EEG marker and cortical atrophy, the correlation between brain areas and the performance to memory tests was investigated in all MCI subgroups. The memory tests were chosen because of their well known greater impairment in MCI subjects who will convert to AD (Dubois et al., 2007; Frisoni, 2012). Our results show no significant memory difference among the groups. This could be a paradoxical outcome. Anyway, it could not be considered a so surprising result, taking in mind the globally mild and early impairment of the whole group of subjects. In other words, when considering strictly the memory performance, the groups are not different. This is probably due to the early and generally MCI, Anyway, despite no significant difference in the memory test scores, when focusing on the relationship between the memory performance and a reliable structural marker, such as the cortical thickness, the MCI group with the higher alpha3/alpha2 power ratio has shown a (negative) correlation between memory tests performance and the cortical thickness, as expected in patients with probable prodromal AD. This result confirms the peculiar nature of this MCI group, showing a clear specificity as regards both the cortical atrophy and the correlated memory performance. Moreover, no other socio-demographical or structural differences were observed in the MCI groups that could explain the correlation analysis results. The cortical areas associated with cortical thinning and those correlated with memory tests performance are only partly overlapping. This could be due to particular nature of the memory domain, underpinning a large number of brain areas. On the other hand, MCI subjects more susceptible to convert to AD could show impairment also in other cognitive domain like as visuospatial attention or in execution and preparation of spatially guided behavior (Ghaem et al., 1997; Leichnetz, 2001; Wenderoth et al., 2005; Cavanna and Trimble, 2006). Of note, the cortical network encompassing precuneus and inferior parietal cortex is deeply involved in visuo-patial abilities (Zhang and Li, 2012). As a speculative interpretation, we could hypothesize that the memory deficits could be due to impaired network underlying the semantic coding of the spatial features of the episodic memory traces. In this view, the atrophy of a specific brain network (more than global volume measures) is more reliable in detecting MCI subjects with prodromal AD. Anyway, the discussion of memory-related brain networks was beyond the scope of the present study. Only a weak negative correlation was found in the middle alpha3/alpha2 EEG power ratio, suggesting a possible degenerative nature of the memory impairment in this group. No significant associations were find in low alpha3/alpha2 power ratio group and in the whole sample. Taken together, these results strengthen the position of the higher alpha3/alpha2 ratio MCI group as at major risk to develop AD.

### IMPLICATIONS AT SYSTEM LEVEL

Klimesch et al. (1996, 2001) have convincingly demonstrated that that the upper alpha band (∽10–13 Hz) specifically reflects encoding memory processes. Recent EEG and magnetoencefalography (MEG) studies have confirmed that a correct functioning of memory, both in encoding and in retrieval, requires the high alpha rhythm desynchronization (or power decrease; Shannon and Weaver, 1949; Fries et al., 2001; Kilner et al., 2005; Wyart and Tallon-Baudry, 2008; Spitzer et al., 2009; Hanslmayr et al., 2010; Staudigl et al., 2010). From a neurophysiological point of view the synchronization (or power increase) of EEG alpha power has been associated with the inhibition timing hypothesis (Shannon and Weaver, 1949) and with poor information transmission, according to he entropy’s theory (Rosen et al., 1980; Hanslmayr et al., 2012). The increases in alpha amplitudes reflect inhibition of cortical brain regions (Folstein et al., 1975; Hughes et al., 1982; Jensen and Mazaheri, 2010). Similarly, the entropy’s theory stated that synchronization is disadvantageous for storing information, as it reduces the flow of information (Rosen et al., 1980). Entropy is a measure of the richness of information encoded in a sequence of events. Applying this concept to the neural networks, it has been demonstrated (Zhang and Li, 2012) that the degree of information that is encoded in neural assemblies increases as a function of desynchronization and decreases as a function of synchronized firing patterns (Norman, 2010; Schneidman et al., 2011). This hypothesis has been confirmed in clinical studies in patients with memory deficits (Kurimoto et al., 2012). as well as during states where there is little cognitive processing (e.g., epileptic seizures or slow wave sleep; Goard and Dan, 2009; Chalk et al., 2010; Zhang and Li, 2012) As regards cognitive impairment due to AD, the typical synaptic loss could prevent the physiological flexibility of brain neural assemblies, impeding the desynchronizing downstream modulation of the brain activity. As a consequence, it could be hypothesized that the disruption of cortical network due to degenerative disease, inducing cortical atrophy, could determine an over synchronization of the brain oscillatory activity. The synchronization state of the high alpha power could prevent the creation of a semantic sensory code and, consequently, of the episodic memory trace (Barlow, 1961; Bialek et al., 1991; Hanslmayr et al., 2009). In previous seminal studies, high alpha frequency has been specifically related to semantic memory processes (Craik, 2002; Moretti et al., 2004). Of note, in subjects with early cognitive decline, the impairment of the semantic features of memory has been recently accepted as a hallmark for the early AD diagnosis (Dubois et al., 2007; Albert et al., 2011). Indeed, according to the new diagnostic criteria of AD, the measurement of sensitivity to semantic cueing can successfully differentiate patients with AD from healthy controls, even when patients are equated to controls on MMSE scores or when disease severity is very mild. Our results are generally in line with this hypothesis, suggesting that increase in power of high alpha brain oscillations reflects a block of information processes. However, the present study goes one step further, linking the increase of high alpha synchronization to the atrophy of a specific brain network, correlated with impairment in memory performances.

### STUDY LIMITATIONS

There are some limitations due to the obvious explorative nature of the present study: (1) further studies are needed to confirm our result on larger samples and applying an appropriate multiple comparison correction; (2) the pattern of cortical thickness should be investigated on the remaining EEG frequency measures; (3) finally the retrospective nature of the study prevented a direct assessment of whether subjects with increase of a3/a2 EEG power ratio will convert to Alzheimer’s or other neurodegenerative disease; (4) the conservative *p* < 0.001 used here is not necessarily sufficient given the number of comparisons. Anyway, given the explorative nature of the study it is plausible a permissive approach in order to avoid to reject possibly interesting results. It remains clear that further studies with less permissive statistical approach are mandatory to confirm results. Of note, the reliability of the results is supported by: (1) the rigorous selection criteria of the subjects; (2) the high statistical threshold (*p* < 0.001) considered; (3) the large size of pixel accounted for the analysis (30 mm^2^); (4) and, finally, the statistical control analysis represented by the correlation of cortical thickness with memory tests.

## CONCLUSION

The present results show that that synchronization (or increase in power) of high alpha is associated with greater cortical atrophy. The greater cortical atrophy is present both considering the whole brain volume and in a peculiar memory-related network, including precuneus and temporo-parietal cortices. The combination of EEG alpha3/alpha2 ratio and cortical thickness measure could be useful for identifying individuals at risk for progression to AD dementia and may be of value in clinical context.

## Conflict of Interest Statement

The authors declare that the research was conducted in the absence of any commercial or financial relationships that could be construed as a potential conflict of interest.
